# Effects of external diaphragm pacing combined with repetitive peripheral magnetic stimulation of the phrenic nerve on respiratory function in stroke patients

**DOI:** 10.3389/fmed.2025.1596850

**Published:** 2025-07-16

**Authors:** Qin Zhang, Chengshuo Wang, Hongfei Cai, Shasha Jin, Qian Wang, Yanxin Fu, Aomeng Xiang, Jingman Qi, Liang Wu, Bin Liu

**Affiliations:** ^1^Department of Sports Rehabilitation, Beijing Xiaotangshan Hospital, Beijing, China; ^2^Tianjin Key Laboratory of Exercise Physiology and Sports Medicine, Institute of Sport, Exercise & Health, Tianjin University of Sport, Tianjin, China; ^3^Department of Neurology, Chengdu Fifth People’s Hospital, Chengdu, Sichuan, China

**Keywords:** stroke, external diaphragmatic pacing, repeated peripheral magnetic stimulation, respiratory function, rehabilitation

## Abstract

**Objectives:**

To investigate the effects of external diaphragmatic pacing (EDP) and repetitive peripheral magnetic stimulation (rPMS) of the phrenic nerve on respiratory function in stroke patients.

**Methods:**

Fifty-four stroke patients were randomly assigned to three groups: an EDP group (*n* = 18), an rPMS group (*n* = 18), and a combined treatment group (*n* = 18). All groups received routine breathing training. Additionally, the EDP group underwent EDP, the rPMS group received repeated peripheral magnetic stimulation of the phrenic nerve, and the combined treatment group received a combination of both interventions. The treatment regimen lasted for 4 weeks. Pulmonary function parameters, including forced vital capacity (FVC), forced expiratory volume in 1 s (FEV_1_), FEV_1_/FVC%, peak expiratory flow (PEF), maximal inspiratory pressure (MIP), and maximal expiratory pressure (MEP), were assessed using a pulmonary function tester. Diaphragmatic thickness (DT) and diaphragmatic excursion (DE) were evaluated via ultrasound imaging, whereas compound muscle action potential (CMAP) amplitude and phrenic nerve conduction time (PNCT) were measured using transcranial magnetic stimulation technology.

**Results:**

Following 4 weeks of treatment, significant improvements were observed in FVC, FEV_1_, PEF, MIP, and MEP across all three groups (all *p < 0.05*). Moreover, the combined treatment group demonstrated significantly greater improvements in FVC, FEV_1_, and MIP compared with either the EDP or rPMS group (*p < 0.05*). DT and DE were also significantly increased in all groups (*p < 0.05*), with more pronounced improvements in the combined treatment group than in the other groups (*p < 0.05*). In all three groups, CMAP amplitude increased significantly, whereas PNCT decreased significantly (*p < 0.05*). Furthermore, the reduction in PNCT was more obvious in the combined treatment group than in either the EDP or rPMS group (*p < 0.05*).

**Conclusion:**

Compared with monotherapy using either EDP or rPMS, combined treatment demonstrates significantly greater efficacy in promoting respiratory function rehabilitation in stroke patients. Additionally, it shows potential advantages in improving phrenic nerve motor conduction.

## 1 Introduction

Stroke induces respiratory muscle weakness and atrophy, thereby increasing the risk of respiratory dysfunction (1). Approximately 40% of people with stroke present with reduced diaphragmatic mobility, forced vital capacity (FVC), and forced expiratory volume in one second (FEV₁), with a decline of up to 50% of the predicted values (2). This pathological change impairs thoracic cavity volume and the function of the affected side chest wall and abdomen, thereby causing diaphragmatic elevation (3). These combined alterations result in respiratory muscle weakness and cough dysfunction, which in turn increase the risk of pneumonia (4). Pulmonary infection is the most prevalent complication in stroke patients, accounting for up to 30% of in-hospital mortality (5). Post-stroke pulmonary rehabilitation has been demonstrated to improve inspiratory muscle strength and endurance, cough efficiency, and cardiopulmonary function (6). Thus, pulmonary rehabilitation for stroke patients is of significant clinical importance.

Currently, multiple rehabilitation therapies are available for respiratory dysfunction, such as respiratory muscle training, proprioceptive neuromuscular facilitation (PNF), respiratory neuromuscular electrical stimulation (RNMES), external diaphragmatic pacing (EDP), and repetitive peripheral magnetic stimulation (rPMS). Specifically, EDP is a therapeutic modality that employs functional electrical stimulation to activate the phrenic nerve, thereby inducing rhythmic diaphragmatic contractions and enabling passive-to-semiautomatic diaphragmatic exercise. This mechanism increases thoracic cavity volume, elevates tidal volume, reduces respiratory muscle tension, alleviates diaphragmatic fatigue, and improves pulmonary ventilation (7, 8). Clinical evidence has also demonstrated that EDP can improve respiratory parameters and increase diaphragmatic thickness (DT) in patients with chronic obstructive pulmonary disease (COPD) (9–12). Recent studies have shown that EDP can improve diaphragmatic function, which is beneficial for the prognosis of patients receiving prolonged mechanical ventilation (MV) (13, 14).

In contrast to EDP, rPMS stimulates receptors without causing skin damage by generating time-varying electromagnetic fields, which induce eddy currents in skeletal muscles and thereby activate the neuromuscular junction. rPMS has emerged as a promising therapeutic modality for addressing cerebral and neural impairments. This approach enhances limb functionality and restores motor performance via neuroplasticity modulation, while maintaining an excellent safety profile with minimal adverse events (15, 16). Considering these unique advantages, rPMS has demonstrated increasing clinical implementation. Current clinical investigations have explored the therapeutic potential of rPMS in sensory and motor rehabilitation (17–20). Furthermore, preclinical evidence has demonstrated that bilateral phrenic nerve magnetic stimulation can induce diaphragmatic contractions and mitigate diaphragmatic atrophy (21). Additionally, studies have shown that combining rPMS with pressure support ventilation generates sufficient minute ventilation (22). Collectively, these findings suggest that this non-invasive approach holds significant promise for clinical application.

In this study, we hypothesized that rPMS combined with EDP treatment was more effective in restoring pulmonary ventilation in stroke patients than EDP or rPMS alone. Therefore, this study aims to analyze the effect of rPMS combined with EDP on the respiratory function of stroke patients.

## 2 Materials and methods

### 2.1 Study design

This study was designed as a randomized, single-blinded clinical trial and performed at a tertiary rehabilitation center. Sample size was calculated using G*Power software (version 3.1.9.7, Germany) based on pre-trial data. The primary study outcome was maximal inspiratory pressure (MIP). With a target power of 0.85 and an alpha level of 0.05, the calculated sample size was 54, accounting for a 20% dropout rate.

Patients were recruited from the Department of Rehabilitation at Beijing Xiaotangshan Hospital between October 2022 and October 2023. Eligible participants were randomly allocated to the EDP group, rPMS group, or combined treatment group by a therapist using a random number table. Assessors and statisticians were blinded to group assignments. Patients received individualized treatment and remained unaware of the study hypothesis; unblinding was prohibited. Due to the nature of interventions, patient blinding was not feasible.

The protocol was approved by the Ethics Committee of Beijing Xiaotangshan Hospital (No. 2021-49) and registered at the Chinese Clinical Trial Registry (ChiCTR2500098532). All participants provided written informed consent.

### 2.2 Participants’ inclusion/exclusion criteria

Patients were included based on the following inclusion criteria: (1) Diagnosis of stroke conforming to the 2015 Chinese Classification of Cerebrovascular Diseases; (2) Imaging-confirmed (cranial MRI or CT) cerebral hemorrhage or infarction within the prior 6 months; (3) Age between 50 and 80 years; (4) Unilateral limb paralysis; (5) Respiratory muscle weakness (≤ 70% of predicted MIP and/or maximal expiratory pressure [MEP]); (6) Ability to understand and follow instructions, with a Montreal Cognitive Assessment score ≥ 21; and (7) Written informed consent provided by the patient or legal guardian.

Patients were excluded if they met any of the following criteria: (1) Brain stem infarction or hemorrhage; (2) History of pulmonary and thoracic-abdominal disease; (3) Severe impairment of vital organ function or critical condition; (4) Presence of pacemakers, cochlear implants, or other metallic implants; (5) History of seizures; (6) Severe cognitive, emotional, or mental disorders precluding cooperation; and (7) Respiratory conditions (such as chronic obstructive pulmonary disease [COPD], tuberculosis, lung cancer) or musculoskeletal issues (such as rib fractures) affecting functional testing.

### 2.3 Procedures

All patients in the three groups received conventional comprehensive therapy, which included nutritional support, pharmacotherapy, occupational therapy, gait training, balance training, and activities of daily living (ADL). Additionally, all patients received conventional breathing physiotherapy for 30 min daily, 5 days per week for 4 weeks, consisting of abdominal breathing, pursed-lip breathing, resistive breathing exercises, breathing control, chest expansion maneuvers, and cough training (23, 24).

**Figure 1:**
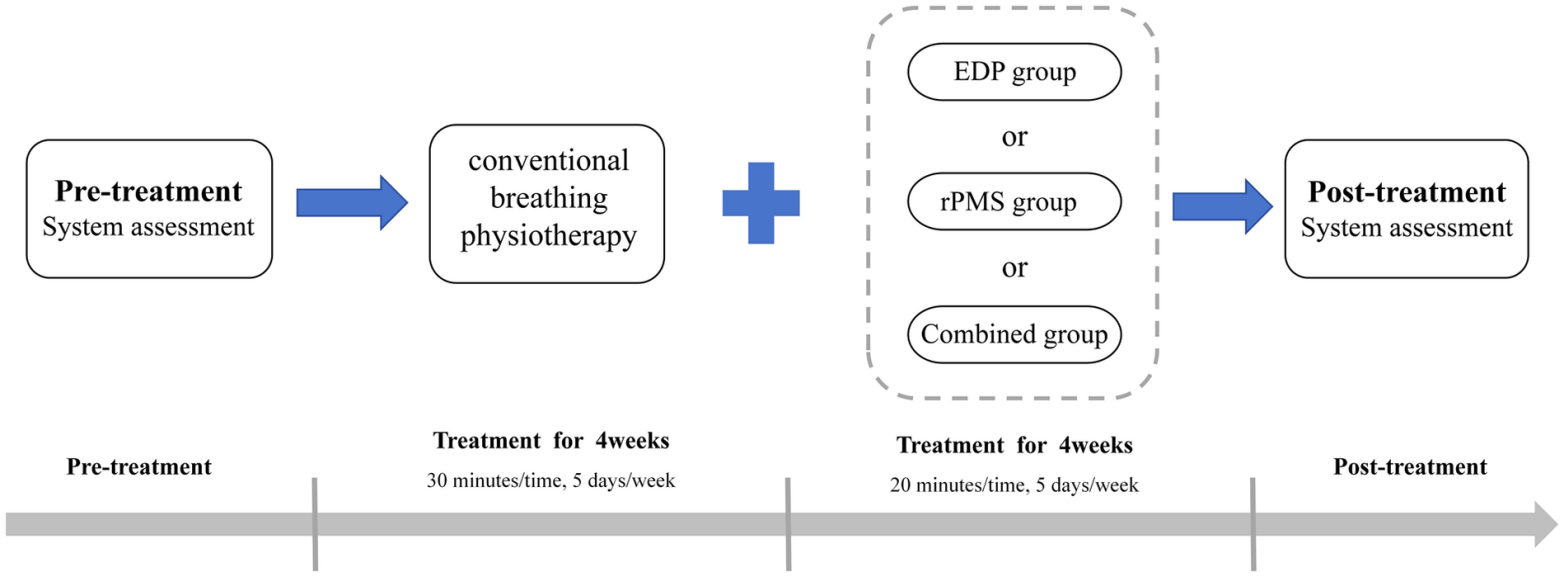
Schematic of the experimental design.

In the EDP group, patients received treatment with a DiaHealth variable frequency portable external diaphragm pacemaker (Shanghai Langyi Medical Devices Co., Ltd., Shanghai, China). Before treatment, the skin was wiped, and the two main electrodes were attached to the inferolateral third of the sternocleidomastoid muscle bilaterally, positioned along the muscle to form an approximate “V” shape. Notably, the main electrodes were not directly placed on the sternocleidomastoid muscle to avoid excessive muscle contraction. The two auxiliary electrodes were applied to the second intercostal space at the midclavicular line, with strict avoidance of cross-placement of bilateral leads. Stimulation parameters: The pacing frequency was set at 15 beats per minute, and the pulse frequency was set at 40 Hz (13), pulse width was set at 200 μs, and pulse intensity ranged from 10 to 15 mA, adjustable according to patient tolerance (25). During stimulation, subjects were instructed to inhale deeply and then exhale naturally. Each treatment session lasted 20 min and was administered 5 days a week for 4 weeks. For details, refer to Figure 1.

In the rPMS group, patients received bilateral phrenic nerve magnetic stimulation during routine respiratory function exercises. Magnetic stimulation was administered using a circular dynamic air-cooled coil (Brainsway Ltd., Jerusalem, Israel; model Intelligent Technology), with a maximum output of 2.2 Tesla. The patient’s Active Motor Threshold (AMT) was measured prior to treatment, with the specific operational procedures detailed below:

(1) The patient was seated with the head in a neutral position. (2) The skin at the electrode sites was carefully wiped with a cotton ball dipped in conductive paste to reduce skin resistance. The recording electrode was placed at the xiphoid process, the reference electrode at the seventh intercostal space of the ipsilateral anterior axillary line, and the ground electrode was attached to the ipsilateral wrist (same side as the recording electrode). (3) The coil was placed in the cortical area where the target muscle motor evoked potential could be collected, which was positioned over Cz as determined by the international 10 to 20 electroencephalograph system (26, 27). (4) The coil was first placed flat over C7 and moved toward C6 in a stepwise manner until the highest reproducible compound muscle action potential (CMAP) amplitude at 100% of the maximum magnetic power output was obtained. The optimal nuchal stimulation site was marked with a pen. (5) AMT determination: Stimulation was applied at the optimal site with gradually reduced intensity. AMT was defined as the minimum stimulation intensity at which, during end-expiration or early inspiration, at least 50% of 10 transcranial magnetic stimulations elicited amplitudes of 200–300 μV. Stimulation parameters: Output intensity: 80–120% of the patient’s AMT (increases controlled within tolerance); Stimulation frequency: 25 Hz (21); Train duration: 1.5 s; Inter-train interval: 3 s (28). The therapist assisted the patient in matching their breathing rate to the stimulation device, using the patient’s natural respiratory rate as a reference. Once treatment commenced, the patient was maintained in a quiet, comfortable state to prevent coil displacement. Each treatment session lasted 20 min and was administered 5 days a week for 4 weeks. For details, refer to Figure 1.

In the combined treatment group, patients received EDP and rPMS therapy, with EDP administered in the morning and rPMS in the afternoon. Each treatment session lasted 20 min and was administered 5 days a week for 4 weeks. For details, refer to Figure 1.

During the treatment, patients were promptly asked by the therapist about any adverse reactions. All adverse events were recorded in the Case Report Form (CRF), and the project manager was immediately notified. The manager collaborated with the patient and physiotherapist to formulate solutions, ensuring patient safety.

### 2.4 Outcome measures

Baseline assessments were conducted 1–3 days prior to intervention initiation, and post-intervention assessments were performed 1–3 days following intervention completion. All assessments were conducted by an assessor blinded to randomization and intervention allocation to maintain confidentiality.

#### 2.4.1 Pulmonary function tests

##### 2.4.1.1 Primary outcome measure

Maximum inspiratory pressure (MIP) is defined as the maximal inspiratory oral pressure generated during maximal inspiratory effort at functional residual capacity (FRC) or residual volume (RV) with airway occlusion. It reflects the comprehensive inspiratory force of all inspiratory muscles and is currently an important non-invasive index for assessing inspiratory muscle function. When MIP falls below 30% of the predicted value, the risk of respiratory failure increases significantly (29). In this study, MIP was used as the primary outcome measure. Studies have reported that stroke patients exhibit an average maximum inspiratory pressure (MIP) of 17–57 cmH_2_O, compared to approximately 100 cmH_2_O in healthy adults (30).

Similar to MIP, maximum expiratory pressure (MEP) is also an important index for evaluating respiratory muscle function. MEP is defined as the maximal oral pressure generated during maximal expiratory effort at total lung capacity (TLC) with airway occlusion, reflecting the collective expiratory force of all expiratory muscles. The average MEP in stroke patients is 25–68 cmH_2_O, versus around 120 cmH_2_O in healthy adults (30).

To accurately measure these parameters, the following procedures were carried out. The patient was positioned supine with the head of the bed elevated at 30°. Pulmonary function parameters, including FVC, forced expiratory volume in one second (FEV₁), FEV₁/FVC %, peak expiratory flow (PEF), maximal inspiratory pressure (MIP), and MEP, were assessed using the Pivot CPX cardiopulmonary exercise testing system. During the assessment, the patient wore a nose clip and maintained a tight seal around the mouthpiece to ensure proper performance of inspiratory and expiratory maneuvers. The mean value of three tests, with an interval of at least 1 min between each test, was calculated as the final result. If any test result deviated by more than 15% from the others, an additional test was conducted.

#### 2.4.2 Diaphragm ultrasound

Before measurement, the ultrasound probe was calibrated according to the manufacturer’s instructions. The patient was placed in the supine position with 30° head elevation and examined using a benchtop ultrasound device (Hitachi, Ltd., Tokyo, Japan; model EZU-HC1C).

**Figure 2:**
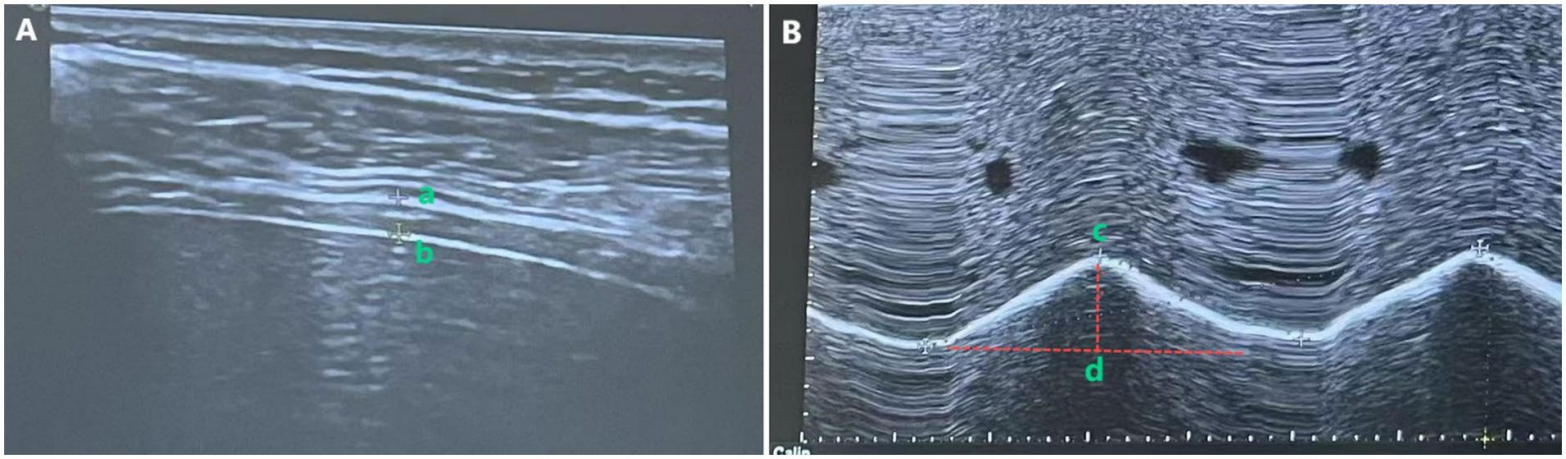
Measurement of diaphragmatic thickness **(A)** and excursion **(B)**.

Diaphragmatic thickness was measured via B-mode ultrasound with a high-frequency linear probe. For the hemiplegic-side diaphragm, DT was assessed with a 2–5 MHz convex array probe. The patient was instructed to maintain quiet breathing. The measurement method of DT is shown in Figure 2A, where the probe was positioned in the 7th to 9th intercostal spaces between the midclavicular line and the anterior axillary line (right diaphragm) or between the anterior axillary line and the midaxillary line (left diaphragm), perpendicular to the chest wall. The diaphragm appeared as a hypoechoic region between the hyperechoic lines of the pleura and peritoneum, as shown in the image recorded as a–b. All measurements were performed in triplicate, and the mean value was recorded.

Diaphragmatic excursion (DE) was measured using M-mode ultrasound. As illustrated in Figure 2B, with the patient in the supine position and 30° head elevation, the ultrasound probe was positioned 2 cm below the costal margin along the right midclavicular line (using the liver as an acoustic window) for the right diaphragm, or 3 cm below the costal margin between the anterior axillary line and the midaxillary line (using the spleen as an acoustic window) for the left diaphragm. In M-mode, the probe was oriented to maximize diaphragmatic motion amplitude, ensuring the sampling line was placed at 90° to the diaphragmatic surface. DE was defined as the vertical displacement between the maximal inspiratory and expiratory points (measured during three consecutive cycles of quiet breathing), recorded as c–d. All measurements were performed in triplicate, and the mean value was used for analysis.

#### 2.4.3 Phrenic nerve motor conduction examination

The assessment was performed using a transcranial magnetic stimulator (Yingzhi Science and Technology Co., Ltd., China) and a VikingQuest electromyography system (Thermo Nicolet Corporation, United States). The patient remained seated with the cervical spine in neutral alignment. Magnetic stimulation was applied through a circular coil (10 cm diameter) centered over the C7 spinous process with its central axis perpendicular to the spinal column. Single-pulse stimuli were delivered at 80% maximal stimulator output (2.2 T) intensity with 30 s interstimulus intervals to avoid habituation effects. Stimulation was synchronized with the initial phase of inspiration. Subsequently, sequential coil repositioning along the cervical neural axis was conducted until reproducible CMAP amplitudes and phrenic nerve conduction time (PNCT) measurements were obtained. If the standard deviation of three consecutive measurements exceeded 15% of the mean value, additional measurements were performed. Finally, three consecutive measurements were averaged for the final analysis.

### 2.5 Statistical analysis

All statistical analyses were performed using SPSS 26.0 (IBM Corp., Armonk, NY, United States). Normality of continuous variables was assessed using the Shapiro–Wilk test (for sample sizes *n* ≤ 50). Normally distributed data were presented as mean (standard deviation, SD), while skewed data were expressed as median with interquartile range (IQR; P25–P75). For within-group comparisons of pre- and post-intervention outcomes, a paired *t*-test was used for normal distributions, and the Wilcoxon signed-rank test for non-normal distributions. Between-group comparisons across the three groups were conducted using one-way analysis of variance (ANOVA) with Levene’s test for homogeneity of variances; when normality or variance assumptions were violated, the Kruskal-Wallis H test was applied. Post-hoc multiple comparisons were performed using Bonferroni’s correction to adjust the significance level. Categorical data were reported as counts (n) and percentages (%), analyzed using the chi-square test or Fisher’s exact test when expected frequencies were < 5. All statistical tests were two-tailed, and significance was set at *p* < 0.05.

## 3 Results

**Figure 3:**
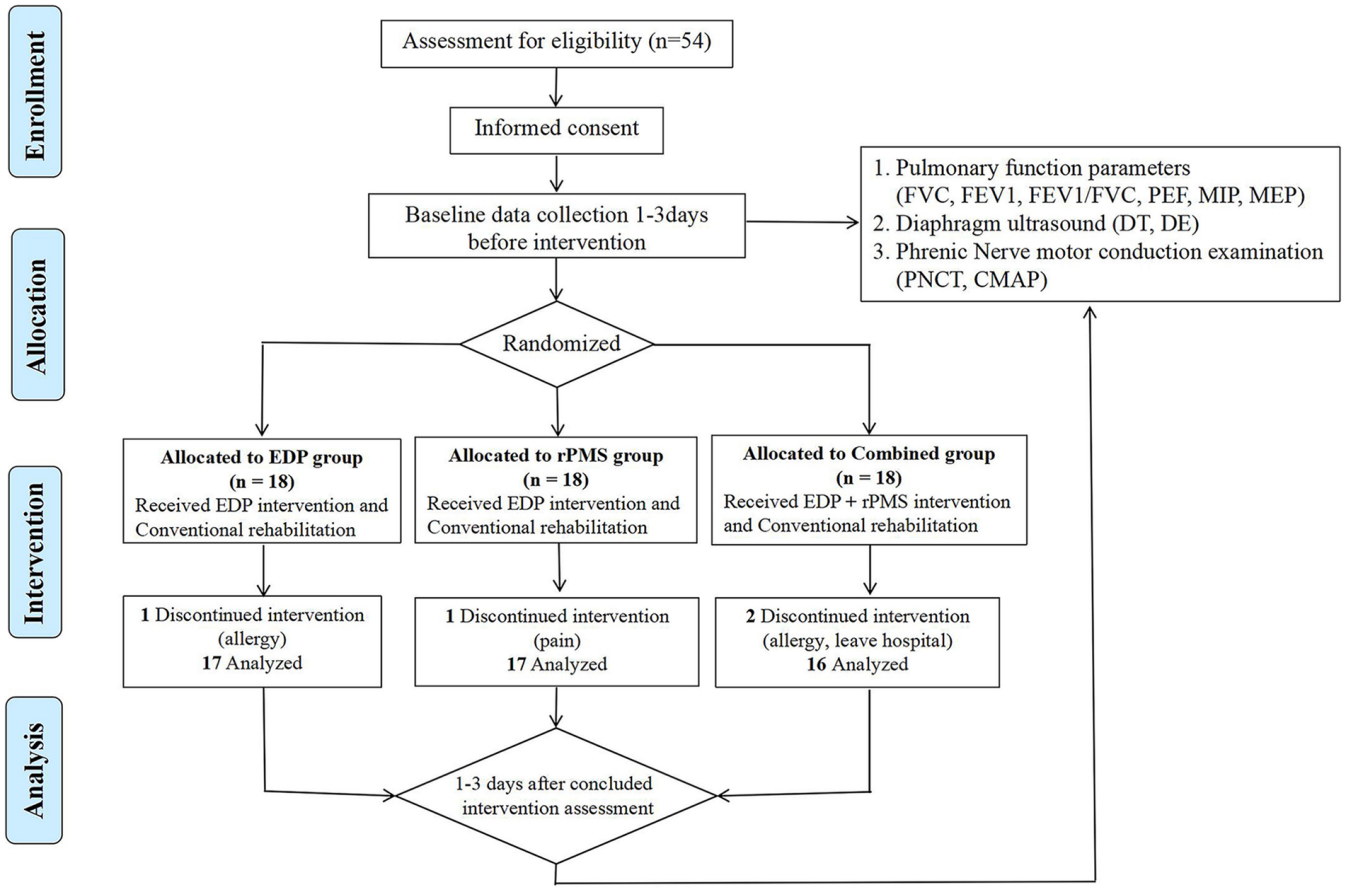
The flowchart of the study.

From October 2022 to October 2023, 54 patients were enrolled and randomly assigned (using a computer-generated random number) to the EDP group (*n* = 18), rPMS group (*n* = 18), or combined treatment group (*n* = 18). During the intervention, one patient in the EDP group withdrew due to a stimulation-site allergy, one patient in the rPMS group discontinued treatment because of neck pain, and the combined treatment group had two patients withdraw due to stimulation-site allergies and one withdraw due to early hospital discharge. All adverse events were mild, resolved completely within 24 h after intervention cessation, and left no residual effects. Consequently, the final analysis included 17 patients in the EDP group, 17 in the rPMS group, and 16 in the combined treatment group. The flowchart is presented as Figure 3.

Baseline demographic and clinical characteristics were comparable across groups, with no significant differences observed (all *p > 0.05*; Table 1), as determined by one-way ANOVA with Levene’s test for continuous variables and chi-square tests for categorical variables.

**Table 1:** Characteristics of demographics at baseline.

Variable	EDP group (*n* = 18)	rPMS group (*n* = 18)	Combined group (*n* = 18)	*P*-value
Age (years) (mean±SD)	60.00 ± 11.98	62.13 ± 10.86	62.03 ± 11.56	0.884
Sex, *n* (%)				0.780
Male, *n* (%)	12 (66.67)	10 (55.56)	11 (61.11)	
Female, *n* (%)	6 (33.33)	8 (44.44)	7 (38.89)	
Course of the disease (days) (mean±SD)	43.19 ± 6.04	42.56 ± 5.88	43.85 ± 6.12	0.453
Type of stroke, *n* (%)				0.816
Ischaemic, *n* (%)	11 (61.11)	12 (66.67)	10 (55.56)	
Haemorrhagic, *n* (%)	7 (38.89)	6 (33.33)	8 (44.44)	
Lesion side, *n* (%)				0.641
Left, *n* (%)	6 (33.33)	5 (38.46)	6 (33.33)	
Right, *n* (%)	12 (66.67)	13 (72.22)	12 (66.67)	

EDP, external diaphragm pacing; rPMS, repetitive peripheral magnetic stimulation; Combined group, EDP combined with rPMS treatment.

### 3.1 Pulmonary function

**Figure 4:**
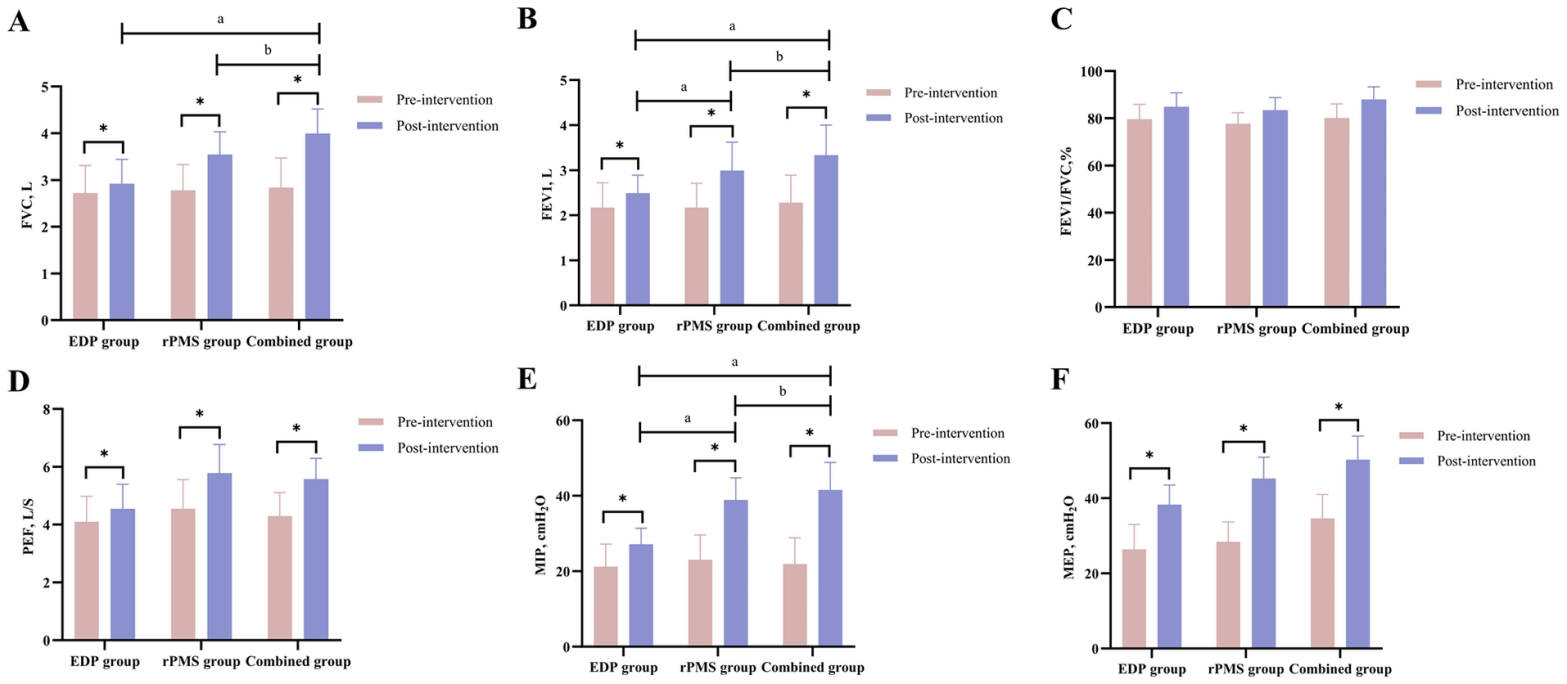
**(A–F)** Pulmonary function (FVC, FEV₁, FEV₁/FVC%, PEF, MIP, MEP) changes in EDP, rPMS, and combined groups. The data are expressed as the mean±SD. ^*^p < 0.05 vs. baseline; ^a^ *p* < 0.05 vs. EDP group; ^b^ *p* < 0.05 vs. rPMS group.

At baseline, no significant intergroup differences were observed in FVC, forced expiratory volume in 1 s (FEV₁), FEV₁/FVC%, PEF, MIP, or MEP among the three groups (*p > 0.05*). Following treatment, all three groups demonstrated significant improvements in FVC, FEV₁, PEF, MIP, and MEP (*p < 0.01*). Notably, the combined treatment group showed significantly greater improvements in FVC (*p = 0.002*), FEV₁ (*p = 0.003*), and MIP (*p = 0.001*) when compared with both the rPMS and EDP groups (*p < 0.05*; Table 2). Mean values and SDs are shown in Figure 4 as bar graphs.

**Table 2:** Intervention-induced variations of respiratory variables in three treatment groups.

Variables	EDP group (*n* = 17)		rPMS group (*n* = 17)		Combined group (*n* = 16)		Intergroup *p*-value (Pre-intervention)	Intergroup *p*-value (post-intervention)
	Pre-intervention	Post-intervention	Pre-intervention	Post-intervention	Pre-intervention	Post-intervention		
FVC, l (mean±SD)	2.72 ± 0.59	2.92 ± 0.52^①^	2.78 ± 0.55	3.54 ± 0.49^①^	2.84 ± 0.63	3.99 ± 0.53^①ab^	0.189	0.031
FEV1, l (mean±SD)	2.17 ± 0.55	2.49 ± 0.40^①^	2.17 ± 0.54	2.99 ± 0.63^①a^	2.28 ± 0.61	3.33 ± 0.67^①ab^	0.130	<0.001
FEV1/FVC, % (mean±SD)	79.58 ± 6.33	84.77 ± 6.04	77.66 ± 4.69	83.38 ± 5.36	80.11 ± 5.92	87.88 ± 5.44	0.939	0.921
PEF, l/s (mean±SD)	4.10 ± 0.88	4.54 ± 0.86^①^	4.55 ± 1.01	5.78 ± 0.89^①^	4.29 ± 0.82	5.57 ± 0.72^①^	0.573	0.389
MIP, cmH_2_O (mean±SD)	21.22 ± 5.98	31.17 ± 4.23^①^	23.07 ± 6.56	38.87 ± 5.92^①a^	21.94 ± 6.93	42.52 ± 7.32^①ab^	0.856	<0.001
MEP, cmH_2_O (mean±SD)	26.37 ± 6.61	38.27 ± 5.24^①^	28.40 ± 5.25	45.23 ± 5.71^①^	34.63 ± 6.33	50.25 ± 6.31^①^	0.128	0.119

FVC, forced vital capacity; FEV1, forced expiratory volume in one second; PEF, peak expiratory flow rate; MIP, maximum inspiratory pressure; MEP, maximum expiratory pressure; EDP, external diaphragm pacing; rPMS, repetitive peripheral magnetic stimulation. ^①^ *p* < 0.05 vs. Pre-intervention; ^a^ *p* < 0.05 vs. EDP group; ^b^ *p* < 0.05 vs. rPMS group.

### 3.2 Diaphragmatic thickness and excursion

**Figure 5:**
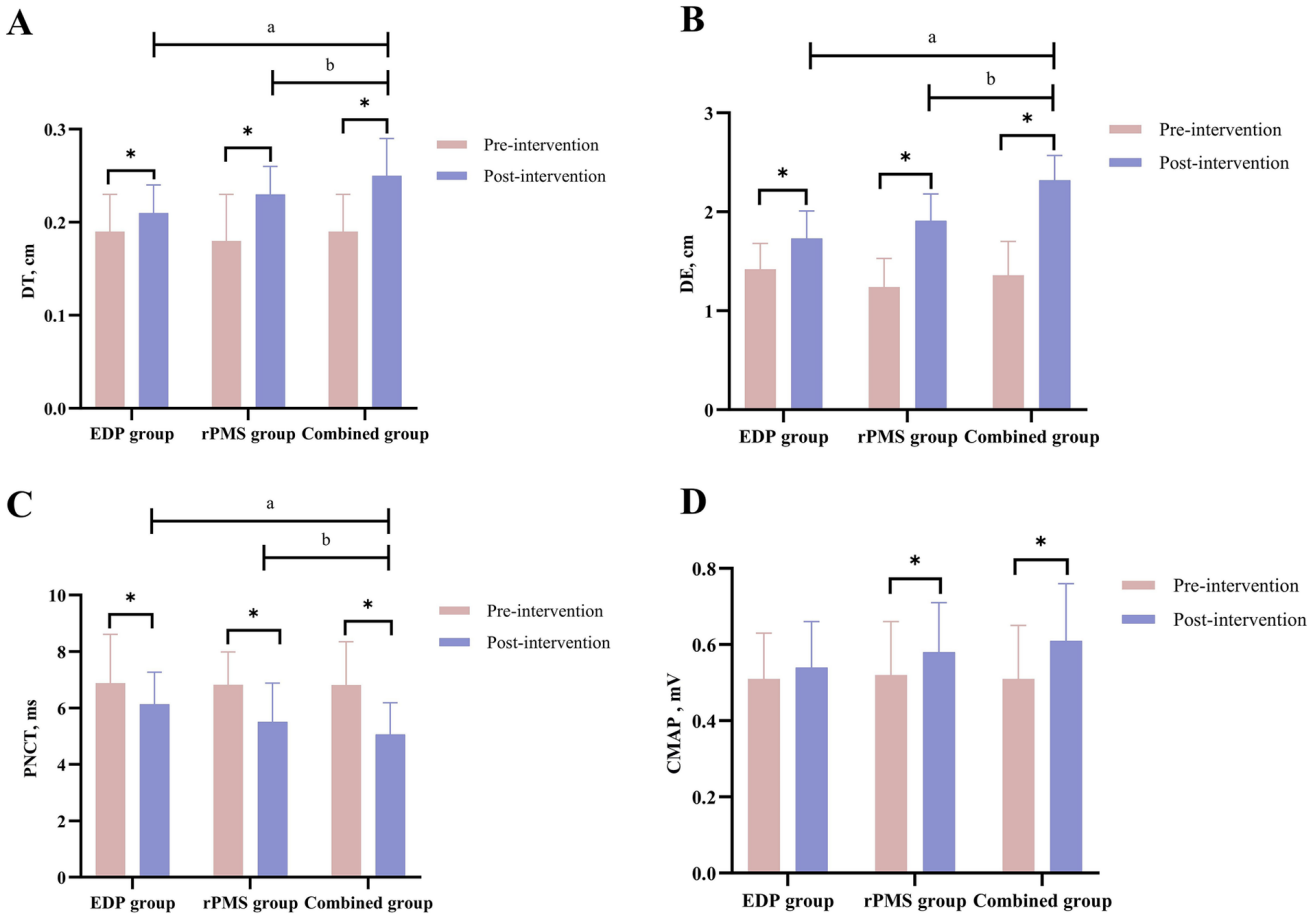
Diaphragmatic thickness (DT) and excursion (DE) results **(A,B)**; PNCT and CMAP of the phrenic nerve **(C,D)**. The data are expressed as the mean±SD. ^*^p < 0.05 vs. baseline; ^a^ *p* < 0.05 vs. EDP group; ^b^ *p* < 0.05 vs. rPMS group.

At baseline, no significant intergroup differences were observed in DT or excursion (DE) among the three groups (*p > 0.05*). Following treatment, all three groups showed significant increases in both DT and DE (*p < 0.01*). The combined treatment group exhibited significantly greater increases in DT (*p = 0.003*) and DE (*p = 0.001*) compared with both the rPMS and EDP groups (*p < 0.05*, Table 3). Figure 5 displays mean values and SDs as bar graphs.

**Table 3:** Changes of diaphragmatic thickness (DT) and excursion (DE) in three intervention groups.

Group	DT, cm		DE, cm		Intragroup *P*-Value (DT)	Intragroup *P*-Value (DM)
	Pre-intervention	Post-intervention	Pre-intervention	Post-intervention		
EDP group (*n* = 17) (mean±SD)	0.19 ± 0.04	0.21 ± 0.03	1.42 ± 0.26	1.73 ± 0.28	0.028	0.003
rPMS group (*n* = 17) (mean±SD)	0.18 ± 0.05	0.22 ± 0.03^a^	1.24 ± 0.29	1.91 ± 0.27^a^	0.001	<0.001
Combined group (*n* = 16) (mean±SD)	0.19 ± 0.04	0.25 ± 0.04^ab^	1.36 ± 0.34	2.32 ± 0.25^ab^	<0.001	<0.001
Intergroup *F*-value	1.185	1.251	0.056	5.485		
Intergroup *P*-value	0.315	0.017	0.945	0.001		

EDP, external diaphragm pacing; rPMS, repetitive peripheral magnetic stimulation. ^a^ *p* < 0.05 vs. EDP group; ^b^ *p* < 0.05 vs. rPMS group.

### 3.3 Phrenic nerve motor conduction assessment

At baseline, no significant intergroup differences were observed in PNCT or CMAP amplitude among the three groups (*p > 0.05*). After treatment, PNCT decreased significantly in both the rPMS and combined treatment groups (*p < 0.01*), with the combined group demonstrating a significantly greater reduction in PNCT (*p = 0.002*) than the rPMS group (*p < 0.05*). Conversely, CMAP amplitude increased significantly across all three groups (*p < 0.01*), but no significant differences were found between the combined group and either the rPMS group (*p = 0.12*) or EDP group (*p = 0.09*; Table 4). Mean values and SDs of PNCT and CMAP are visualized in Figure 5.

**Table 4:** Comparison of PNCT and CMAP in three groups.

Group	PNCT, ms		CMAP, mV		Intragroup *P*-Value (PNCT)	Intragroup *P*-Value (CMAP)
	Pre-intervention	Post-intervention	Pre-intervention	Post-intervention		
EDP group (*n* = 17) (mean±SD)	6.88 ± 1.73	6.13 ± 1.14	0.51 ± 0.12	0.54 ± 0.12	0.061	0.025
rPMS group (*n* = 17) (mean±SD)	6.82 ± 1.16	5.51 ± 1.37^a^	0.52 ± 0.14	0.59 ± 0.13	<0.001	0.017
Combined group (*n* = 16) (mean±SD)	6.81 ± 1.54	5.06 ± 1.12^ab^	0.51 ± 0.14	0.63 ± 0.15	<0.001	<0.001
Intergroup *F*-value	0.172	7.188	0.063	0.082		
Intergroup *P*-value	0.843	0.001	0.939	0.921		

EDP, external diaphragm pacing; rPMS, repetitive peripheral magnetic stimulation; PNCT, phrenic nerve conduction time; CMAP, phrenic nerve complex muscle action potential. ^a^ *p* < 0.05 vs. EDP group; ^b^ *p* < 0.05 vs. rPMS group.

## 4 Discussion

This study investigated the effects of EDP combined with rPMS on respiratory function in stroke patients. Following 4 weeks of intervention, the combined treatment group demonstrated significantly greater improvements in pulmonary function, DT, and DE when compared with either EDP or rPMS monotherapy. These results indicate that adding rPMS to EDP therapy enhances respiratory function rehabilitation in stroke patients, thereby supporting our study hypothesis.

Notably, the combined group also exhibited a significant reduction in PNCT and an increase in CMAP amplitude. These findings suggest that EDP-rPMS combination therapy promotes motor function recovery of both the phrenic nerve and diaphragm.

The diaphragm serves as the primary inspiratory muscle, with its contraction essential for respiratory mechanics and pulmonary ventilation (31). Brain injuries can disrupt phrenic nerve innervation, leading to reduced DE and diaphragmatic atrophy (32, 33). After a stroke, the diaphragm on the affected side exhibits significant thinning compared with the healthy side, with diaphragmatic dysfunction reported in up to 51.7% of acute stroke patients (34). Pulmonary function decline in these patients is multifactorial: diaphragmatic dysfunction, reduced exercise capacity, impaired thoracic expansion, respiratory muscle weakness, respiratory muscle cell loss, and muscle fibrosis (35). Moreover, compromised diaphragmatic function and exercise intolerance further reduce lung compliance and oxygen delivery, exacerbating pulmonary dysfunction and reducing cardiopulmonary reserve. This cascade of effects predisposes to secondary complications (e.g., pneumonia, atelectasis), severely hindering patient recovery (36).

EDP elicits diaphragmatic contraction by external electrical stimulation of the phrenic nerve, recruiting diaphragmatic motor units to enhance type-specific fiber functions while maintaining muscle fiber proportions. This mechanism improves diaphragmatic endurance and strength, induces regular near-physiological respiratory movements, and enhances respiratory function and pulmonary ventilation (35). Our study revealed that 4 weeks of EDP treatment significantly improved patients’ FVC, FEV_1_, PEF, MIP, and MEP. These findings thus demonstrate that EDP enhances lung capacity and respiratory muscle strength by improving diaphragmatic contractility.

A meta-analysis of extracorporeal diaphragm pacing (EDP) combined with conventional rehabilitation for chronic obstructive pulmonary disease (COPD) has shown that EDP improves FVC, FEV₁, and PEF (11). Another multicenter, prospective randomized controlled trial (RCT) with 120 COPD patients reported that 4 weeks of EDP therapy significantly increased FEV₁ and FEV₁/FVC ratios compared with baseline (12). Consistently, Zhu et al. (37) demonstrated that a 4-week EDP intervention in stroke patients improved respiratory parameters, including FVC, FEV₁, PEF, and MIP. All the above results are consistent with our findings.

In recent years, advancements in magnetic stimulation technology have driven increasing attention to rPMS. This technology represents an innovative non-invasive neurorehabilitation approach, regulating peripheral motor nerves through direct axonal activation and indirect neural pathways (38). Neurophysiological studies have confirmed that both cortical and cervical magnetic stimulation effectively evoke phrenic nerve-derived motor-evoked potentials and CMAPs, establishing the neurobiological foundation for rPMS-mediated respiratory function enhancement via modulation of phrenic nerve excitability and diaphragmatic motor unit recruitment (39). Current preclinical and clinical evidence supports its applications in respiratory rehabilitation: phrenic nerve magnetic stimulation in healthy volunteers elicits diaphragmatic contractions with sufficient intensity and duration to support ventilation, demonstrating safety and feasibility (14, 40).

This study demonstrated that 4 weeks of rPMS treatment significantly improved patients’ FVC, FEV_1_, PEF, MIP, and MEP, indicating that rPMS enhances respiratory function and respiratory muscle strength in stroke patients. Jung et al. (41) reported a 50% extubation success rate (*n* = 40, *p < 0.05*) in ICU patients receiving phrenic nerve magnetic stimulation, with a concomitant significant improvement in MIP. These findings suggest that rPMS enhances respiratory muscle strength in intubated patients and improves extubation outcomes, supporting its clinical utility in reducing ventilator dependency. Similarly, a multicenter prospective RCT allocated 40 mechanically ventilated patients to either a phrenic nerve magnetic stimulation group or a control group. After 10 days, the intervention group significantly increased in diaphragm thickness and MIP compared with the control group (all *p < 0.05*), suggesting that rPMS alleviates ventilator-induced diaphragmatic atrophy and enhances respiratory muscle strength (42). Notably, however, current research on the effects of rPMS on respiratory function remains limited, particularly in stroke populations. This highlights the need for large-scale, longitudinal investigations to validate these findings and define optimal treatment protocols.

Ultrasound examination, a non-invasive modality, enables convenient and accurate assessment of diaphragmatic anatomy and function. Owing to these advantages, this study utilized ultrasound to evaluate diaphragmatic function in post-stroke patients. Diaphragmatic thickness and excursion are well-established markers of diaphragmatic contractility, with DT reflecting diaphragmatic atrophy degree (reduced thickness indicates muscle wasting) and DE indicating the diaphragm’s activity capacity (43).

The study demonstrated significant improvements in both DT and DE in all three patient groups post-treatment (*p < 0.01*). Notably, the combined treatment group exhibited significantly greater improvements in DT and DE than the EDP and rPMS groups (*p < 0.05*). Overall, these findings suggest that both EDP and rPMS promote diaphragmatic contraction, as evidenced by increased muscle thickness and excursion, with combination therapy conferring superior benefits.

Our results are consistent with prior evidence. Zhu et al. (37) reported that EDP combined with respiratory training enhanced DT in stroke patients. Beaulieu et al. (17) observed a 15% increase in diaphragm thickness following 48 h of EDP in ventilated patients. Additionally, Sotak et al. (36) demonstrated that 48 h of phrenic nerve stimulation not only reduced diaphragmatic atrophy but also increased diaphragm thickness in patients on mechanical ventilation. Schreiber et al. (42) further showed that rPMS alleviated ventilator-induced diaphragmatic atrophy and enhanced respiratory muscle strength. While several studies have confirmed the safety and feasibility of phrenic nerve magnetic stimulation (44–46), the current evidence base remains limited, necessitating further exploration of its underlying mechanisms.

A key innovative finding of this study is that the post-treatment (4 weeks) PNCT significantly decreased in both the rPMS group and the combined therapy group (*p < 0.01*), with the combined group demonstrating significantly greater efficacy than the rPMS group (*p < 0.05*). Furthermore, significant increases in CMAP amplitude were observed across all three groups post-treatment (*p < 0.01*).

The primary parameters of Phrenic nerve conduction (PNC) are latency and amplitude. Latency denotes the time required for nerve impulses to travel through fast-conducting axonal fibers from the stimulation site to the recording site, reflecting the functional status of the nerve myelin sheath; The amplitude reflects the number of nerve fibers measured and the degree of synchronous excitation, demonstrating a direct proportionality to the number of excited muscle fibers (47). Diaphragm MEP induced by transcranial magnetic stimulation was used to evaluate diaphragmatic function in stroke patients, revealing prolonged PNCT and reduced amplitude in hemiplegic patients. Notably, the amplitude of hemiplegic patients was significantly lower than that of healthy patients, suggesting that the cortical-diaphragmatic pathway was damaged (2). A study in COPD patients using EDP demonstrated shortened PNCT and increased CMAP amplitude post-intervention (48), findings that are consistent with our results.

Furthermore, a comparative study of phrenic nerve magnetic versus electrical stimulation revealed that magnetic stimulation resulted in shorter PNCT than electrical stimulation, with posterior cervical magnetic stimulation-induced PNCT increasing as stimulation intensity decreased (49). This suggests superior phrenic nerve depolarization efficacy with magnetic stimulation, potentially explaining the observed advantage of rPMS over EDP in improving PNCT in our study.

It should be pointed out that the results of this study showed that the improvement in FVC, FEV₁, MIP, DT, DE, and PNCT in the combination group was better than that in the EDP or rPMS group. This may be attributed to diaphragmatic fatigue proneness. Salmons et al. (50) found that stimulation with >40,000 pulses per session could cause muscle fatigue, whereas stimulation with <40,000 pulses showed sufficient fatigue resistance and muscle strength. In this study, EDP was delivered at 40 Hz for 20 min daily, with each stimulation delivering >40,000 pulses, thereby inducing diaphragmatic fatigue. By contrast, the rPMS group received 25 Hz stimulation for 20 min daily, with <40,000 pulses per session, within which the diaphragm was less prone to fatigue. While 40 Hz EDP stimulation induced diaphragmatic fatigue in this study, prior research has shown that EDP not only improved pulmonary mechanics, promoted uniform ventilation distribution, and enhanced gas exchange (9), but also stimulated diaphragmatic contraction, recruited motor units, maintained muscle fiber proportions, and prevented diaphragmatic atrophy (10). Thus, EDP treatment remained effective compared with baseline. The combined treatment group received twice-daily stimulations (morning EDP and afternoon rPMS), a scheduling strategy that minimized diaphragmatic fatigue while preventing atrophy by balancing stimulation intensity with recovery intervals. These mechanisms likely underlie the superior efficacy of combined therapy.

Significantly, the available evidence regarding this topic remains scarce. As a clinical observational study, this investigation has not yet elucidated the underlying mechanisms, warranting further mechanistic exploration through basic research studies.

## 5 Limitations

The current research has several notable limitations. First, the stimulation frequencies of EDP and rPMS were not unified, which might introduce variability in the results. This is primarily because current research on rPMS remains limited, so this study could only adopt treatment parameters proven safe and effective in prior studies, with mechanistic investigations being even scarcer. Future studies should standardize stimulation frequencies while exploring the underlying mechanisms simultaneously. Second, the relatively small sample size and single-center design may limit the generalizability of our findings, highlighting the need for large-scale multicenter trials to validate these results. Third, the 4-week intervention period might be insufficient, as some indicators may not show significant changes. Extending the intervention duration is necessary to observe long-term effects. Finally, the lack of long-term follow-up evaluation obscures the sustained benefits of combined respiratory rehabilitation. Relevant mechanistic research should be conducted to explore the therapeutic potential of these interventions.

## 6 Conclusion

Compared with monotherapy using either EDP or rPMS, combined treatment demonstrated significantly greater efficacy in promoting respiratory function rehabilitation in stroke patients. Additionally, it showed potential advantages in improving phrenic nerve motor conduction.

## Data Availability

The original contributions presented in the study are included in the article/Supplementary material, further inquiries can be directed to the corresponding authors.

## References

[1] Britto RR Rezende NR Marinho KC Torres JL Parreira VF Teixeira-Salmela LF. Inspiratory muscular training in chronic stroke survivors: a randomized controlled trial. Arch Phys Med Rehabil. (2011) 92:184–90. doi: 10.1016/j.apmr.2010.09.029, PMID: 21272713

[2] Khedr EM El Shinawy O Khedr T Abdel Aziz Ali Y Awad EM. Assessment of corticodiaphragmatic pathway and pulmonary function in acute ischemic stroke patients. Eur J Neurol. (2000) 7:509–16. doi: 10.1046/j.1468-1331.2000.00104.x, PMID: 11054135

[3] Lanini B Bianchi R Romagnoli I Coli C Binazzi B Gigliotti F. Chest wall kinematics in patients with hemiplegia. Am J Respir Crit Care Med. (2003) 168:109–13. doi: 10.1164/rccm.200207-745OC, PMID: 12714347

[4] De Troyer A Zegers De Beyl D Thirion M. Function of the respiratory muscles in acute hemiplegia. Am Rev Respir Dis. (1981) 123:631–2. doi: 10.1164/arrd.1981.123.6.631, PMID: 7271058

[5] Ward K Seymour J Steier J Jolley CJ Polkey MI Kalra L. Acute ischaemic hemispheric stroke is associated with impairment of reflex in addition to voluntary cough. Eur Respir J. (2010) 36:1383–90. doi: 10.1183/09031936.00010510, PMID: 20413536

[6] Fabero-Garrido R Del Corral T Angulo-Diaz-Parreno S Plaza-Manzano G Martin-Casas P Cleland JA. Respiratory muscle training improves exercise tolerance and respiratory muscle function/structure post-stroke at short term: a systematic review and meta-analysis. Ann Phys Rehabil Med. (2022) 65:101596. doi: 10.1016/j.rehab.2021.101596, PMID: 34687960

[7] Chen Y Li P Wang J Wu W Liu X. Assessments and targeted rehabilitation therapies for diaphragmatic dysfunction in patients with chronic obstructive pulmonary disease: a narrative review. Int J Chron Obstruct Pulmon Dis. (2022) 17: 457–73. doi: 10.2147/COPD.S338583 PMID: 35273448 PMC8902058

[8] Ahn B Beaver T Martin T Hess P Brumback BA Ahmed S. Phrenic nerve stimulation increases human diaphragm fiber force after cardiothoracic surgery. Am J Respir Crit Care Med. (2014) 190:837–9. doi: 10.1164/rccm.201405-0993LE, PMID: 25271750 PMC4299610

[9] Xu Y Yang D Lu B Zhang Y Ren L Shen H. Efficacy of aerobic training and resistance training combined with external diaphragm pacing in patients with chronic obstructive pulmonary disease: a randomized controlled study. Clin Rehabil. (2023) 37:1479–91. doi: 10.1177/02692155231172005, PMID: 37122164

[10] Lee AHY Snowden CP Hopkinson NS Pattinson KTS. Pre-operative optimisation for chronic obstructive pulmonary disease: a narrative review. Anaesthesia. (2021) 76:681–94. doi: 10.1111/anae.15187, PMID: 32710678

[11] Jiang L Sun P Li P Wu W Wang Z Liu X. Effects of external diaphragm pacing combined with conventional rehabilitation therapies in patients with chronic obstructive pulmonary disease: a systematic review and meta-analysis. Ther Adv Respir Dis. (2023) 17:1611169882. doi: 10.1177/17534666231218086, PMID: 38140896 PMC10748909

[12] Zhao Z Sun W Zhao X Wang X Lin Y Zhang S. Stimulation of both inspiratory and expiratory muscles versus diaphragm-only paradigm for rehabilitation in severe chronic obstructive pulmonary disease patients: a randomized controlled pilot study. Eur J Phys Rehabil Med. (2022) 58:487–96. doi: 10.23736/S1973-9087.22.07185-4, PMID: 35102732 PMC9980572

[13] Bao Q Chen L Chen X Li T Xie C Zou Z. The effects of external diaphragmatic pacing on diaphragm function and weaning outcomes of critically ill patients with mechanical ventilation: a prospective randomized study. Ann Transl Med. (2022) 10:1100. doi: 10.21037/atm-22-4145, PMID: 36388825 PMC9652530

[14] Panelli A Grimm AM Krause S Verfuss MA Ulm B Grunow JJ. Noninvasive electromagnetic phrenic nerve stimulation in critically ill patients: a feasibility study. Chest. (2024) 166:502–10. doi: 10.1016/j.chest.2024.02.035, PMID: 38403186 PMC11443241

[15] Sakai K Yasufuku Y Kamo T Ota E Momosaki R. Repetitive peripheral magnetic stimulation for patients after stroke. Stroke. (2020) 51:e105–6. doi: 10.1161/STROKEAHA.120.029373 32312219

[16] Abe G Oyama H Liao Z Honda K Yashima K Asao A. Difference in pain and discomfort of comparable wrist movements induced by magnetic or electrical stimulation for peripheral nerves in the dorsal forearm. Med Devices (Auckl). (2020) 13:439–47. doi: 10.2147/MDER.S271258, PMID: 33376417 PMC7755354

[17] Beaulieu LD Schneider C. Effects of repetitive peripheral magnetic stimulation on normal or impaired motor control. A review. Neurophysiol Clin. (2013) 43:251–60. doi: 10.1016/j.neucli.2013.05.003 24094911

[18] Fujimura K Kagaya H Itoh R Endo C Tanikawa H Maeda H. Repetitive peripheral magnetic stimulation for preventing shoulder subluxation after stroke: a randomized controlled trial. Eur J Phys Rehabil Med. (2024) 60:216–24. doi: 10.23736/S1973-9087.24.08264-9, PMID: 38483332 PMC11114152

[19] Jiang Y Zhang D Zhang J Hai H Zhao Y Ma Y. A randomized controlled trial of repetitive peripheral magnetic stimulation applied in early subacute stroke: effects on severe upper-limb impairment. Clin Rehabil. (2022) 36:693–702. doi: 10.1177/02692155211072189, PMID: 34985366

[20] Beaulieu L Masse-Alarie H Camire-Bernier S Ribot-Ciscar E Schneider C. After-effects of peripheral neurostimulation on brain plasticity and ankle function in chronic stroke: the role of afferents recruited. Neurophysiol Clin. (2017) 47:275–91. doi: 10.1016/j.neucli.2017.02.003 28314519

[21] Boyle KGPJ Eichenberger PA Schon P Spengler CM. Inspiratory response and side-effects to rapid bilateral magnetic phrenic nerve stimulation using differently shaped coils: implications for stimulation-assisted mechanical ventilation. Respir Res. (2022) 23:357. doi: 10.1186/s12931-022-02251-y, PMID: 36528761 PMC9758474

[22] Panelli A Bartels HG Krause S Verfuss MA Grimm AM Carbon NM. First non-invasive magnetic phrenic nerve and diaphragm stimulation in anaesthetized patients: a proof-of-concept study. Intensive Care Med Exp. (2023) 11:20. doi: 10.1186/s40635-023-00506-6, PMID: 37081235 PMC10118662

[23] Deme S Lamba D Alamer A Melese H Ayhualem S Imeru D. Effectiveness of respiratory muscle training on respiratory muscle strength, pulmonary function, and respiratory complications in stroke survivors: a systematic review of randomized controlled trials. Degener Neurol Neuromuscul Dis. (2022) 12:75–84. doi: 10.2147/DNND.S348736, PMID: 35411199 PMC8994559

[24] Cao H Chen X Ren X Chen Z Liu C Ni J. Repetitive transcranial magnetic stimulation combined with respiratory muscle training for pulmonary rehabilitation after ischemic stroke-a randomized, case-control study. Front Aging Neurosci. (2022) 14:1006696. doi: 10.3389/fnagi.2022.1006696, PMID: 36212033 PMC9537039

[25] Chen X Li C Zeng L Rong T Lin P Wang Q. Comparative efficacy of different combinations of acapella, active cycle of breathing technique, and external diaphragmatic pacing in perioperative patients with lung cancer: a randomised controlled trial. BMC Cancer. (2023) 23:282. doi: 10.1186/s12885-023-10750-4, PMID: 36978035 PMC10053339

[26] Acharya JN Hani A Cheek J Thirumala P Tsuchida TN. American clinical neurophysiology society guideline 2: guidelines for standard electrode position nomenclature. J Clin Neurophysiol. (2016) 33:308–11. doi: 10.1097/WNP.0000000000000316, PMID: 27482794

[27] Khedr EM Trakhan MN. Localization of diaphragm motor cortical representation and determination of corticodiaphragmatic latencies by using magnetic stimulation in normal adult human subjects. Eur J Appl Physiol. (2001) 85:560–6. doi: 10.1007/s004210100504, PMID: 11718285

[28] Menezes KK Nascimento LR Ada L Polese JC Avelino PR Teixeira-Salmela LF. Respiratory muscle training increases respiratory muscle strength and reduces respiratory complications after stroke: a systematic review. J Physiother. (2016) 62:138–44. doi: 10.1016/j.jphys.2016.05.014, PMID: 27320833

[29] Illi SK Held U Frank I Spengler CM. Effect of respiratory muscle training on exercise performance in healthy individuals: a systematic review and meta-analysis. Sports Med. (2012) 42:707–24. doi: 10.1007/BF03262290, PMID: 22765281

[30] Polese JC Pinheiro MB Faria CDCM Britto RR Parreira VF Teixeira-Salmela LF. Strength of the respiratory and lower limb muscles and functional capacity in chronic stroke survivors with different physical activity levels. Braz J Phys Ther. (2013) 17:487–93. doi: 10.1590/S1413-35552012005000114, PMID: 24173350

[31] Greising SM Ottenheijm CAC O'Halloran KD Barreiro E. Diaphragm plasticity in aging and disease: therapies for muscle weakness go from strength to strength. J Appl Physiol (1985). (2018) 125:243–53. doi: 10.1152/japplphysiol.01059.2017, PMID: 29672230 PMC6139508

[32] Kim M Lee K Cho J Lee W. Diaphragm thickness and inspiratory muscle functions in chronic stroke patients. Med Sci Monit. (2017) 23:1247–53. doi: 10.12659/msm.900529, PMID: 28284044 PMC5358861

[33] Park G Kim S Kim YW Jo KW Lee EJ Kim YM. Decreased diaphragm excursion in stroke patients with dysphagia as assessed by m-mode sonography. Arch Phys Med Rehabil. (2015) 96:114–21. doi: 10.1016/j.apmr.2014.08.019, PMID: 25234476

[34] Catala-Ripoll JV Monsalve-Naharro JA Hernandez-Fernandez F. Incidence and predictive factors of diaphragmatic dysfunction in acute stroke. BMC Neurol. (2020) 20:79. doi: 10.1186/s12883-020-01664-w, PMID: 32138697 PMC7057624

[35] Liu Z Wang L Zhao L Pang Y Liu Y Xu W. Clinical effect of pulmonary rehabilitation combined with diaphragm pacemaker therapy in the treatment of severely ill patients with mechanical ventilation. Int J Rehabil Res. (2022) 45:195–200. doi: 10.1097/MRR.0000000000000535 35929569

[36] Sotak M Roubik K Henlin T Tyll T. Phrenic nerve stimulation prevents diaphragm atrophy in patients with respiratory failure on mechanical ventilation. BMC Pulm Med. (2021) 21:314. doi: 10.1186/s12890-021-01677-2, PMID: 34625059 PMC8500254

[37] Zhu T Jin H Liu S Zhu H Wang J. Effects of extracorporeal diaphragm pacing combined with inspiratory muscle training on respiratory function in people with stroke: a randomized controlled trial. Neurol Res. (2024) 46:727–34. doi: 10.1080/01616412.2024.2347133, PMID: 38661091

[38] Chang C Chen C Chen R Chen H Chen C Chung C. Synergistic efficacy of repetitive peripheral magnetic stimulation on central intermittent theta burst stimulation for upper limb function in patients with stroke: a double-blinded, randomized controlled trial. J Neuroeng Rehabil. (2024) 21:49. doi: 10.1186/s12984-024-01341-w, PMID: 38589875 PMC11000298

[39] Spiesshoefer J Henke C Herkenrath S Randerath W Schneppe M Young P. Electrophysiological properties of the human diaphragm assessed by magnetic phrenic nerve stimulation: normal values and theoretical considerations in healthy adults. J Clin Neurophysiol. (2019) 36:375–84. doi: 10.1097/WNP.0000000000000608, PMID: 31145172

[40] Starkova EY Vladimirova NN Tsvetkova EM Litau VY Melnikova EA. Electromagnetic stimulation in diaphragm dysfunction: repetitive peripheral magnetic stimulation as a method of choice during the rehabilitation period after stroke (literature review). Vopr Kurortol Fizioter Lech Fiz Kult. (2024) 101:57–65. doi: 10.17116/kurort202410105157, PMID: 39487620

[41] Jung B Moury PH Mahul M de Jong A Galia F Prades A. Diaphragmatic dysfunction in patients with icu-acquired weakness and its impact on extubation failure. Intensive Care Med. (2016) 42:853–61. doi: 10.1007/s00134-015-4125-2, PMID: 26572511

[42] Schreiber AF Subira C Sklar M Santos M Ko M Panelli A. Multi-center randomized superiority clinical trial in the early phase of mechanically ventilated patients to preserve diaphragm thickness using non-invasive magnetic phrenic nerve stimulation: stimit activator 1 pivotal trial. Trials. (2025) 26:202. doi: 10.1186/s13063-025-08838-2, PMID: 40500715 PMC12153206

[43] Theerawit P Eksombatchai D Sutherasan Y Suwatanapongched T Kiatboonsri C Kiatboonsri S. Diaphragmatic parameters by ultrasonography for predicting weaning outcomes. BMC Pulm Med. (2018) 18:175. doi: 10.1186/s12890-018-0739-9, PMID: 30470204 PMC6251135

[44] Adler D Gottfried SB Bautin N Mirkovic T Schmidt M Raux M. Repetitive magnetic stimulation of the phrenic nerves for diaphragm conditioning: a normative study of feasibility and optimal settings. Appl Physiol Nutr Metab. (2011) 36:1001–8. doi: 10.1139/h11-095, PMID: 22014178

[45] Mills GH Kyroussis D Hamnegard CH Wragg S Moxham J Green M. Unilateral magnetic stimulation of the phrenic nerve. Thorax. (1995) 50:1162–72. doi: 10.1136/thx.50.11.1162, PMID: 8553272 PMC475088

[46] Rafferty GF Greenough A Dimitriou G Kavadia V Laubscher B Polkey MI. Assessment of neonatal diaphragm function using magnetic stimulation of the phrenic nerves. Am J Respir Crit Care Med. (2000) 162:2337–40. doi: 10.1164/ajrccm.162.6.2004019, PMID: 11112160

[47] Markand ON Kincaid JC Pourmand RA Moorthy SS King RD Mahomed Y. Electrophysiologic evaluation of diaphragm by transcutaneous phrenic nerve stimulation. Neurology. (1984) 34:604–14. doi: 10.1212/wnl.34.5.604, PMID: 6324032

[48] Na EH Han SJ Yoon TS. Effect of active pulmonary rehabilitation on pulmonary function in patients with brain lesion. NeuroRehabilitation. (2014) 35:459–66. doi: 10.3233/NRE-141137, PMID: 25248446

[49] Similowski T Mehiri S Duguet A Attali V Straus C Derenne JP. Comparison of magnetic and electrical phrenic nerve stimulation in assessment of phrenic nerve conduction time. J Appl Physiol. (1997) 82:1190–9. doi: 10.1152/jappl.1997.82.4.1190, PMID: 9104856

[50] Salmons S. Adaptive change in electrically stimulated muscle: a framework for the design of clinical protocols. Muscle Nerve. (2009) 40:918–35. doi: 10.1002/mus.21497, PMID: 19902542

