# Supplementary material for: Effects of external diaphragm pacing combined with repetitive peripheral magnetic stimulation of the phrenic nerve on respiratory function in stroke patients
Source: Front Med (Lausanne). 2025 Jul 16;12:1596850. doi: 10.3389/fmed.2025.1596850 (PMC12307151; doi:10.3389/fmed.2025.1596850)
Supplement: Supplementary file 1 [file Supplementary_file_1.pdf]

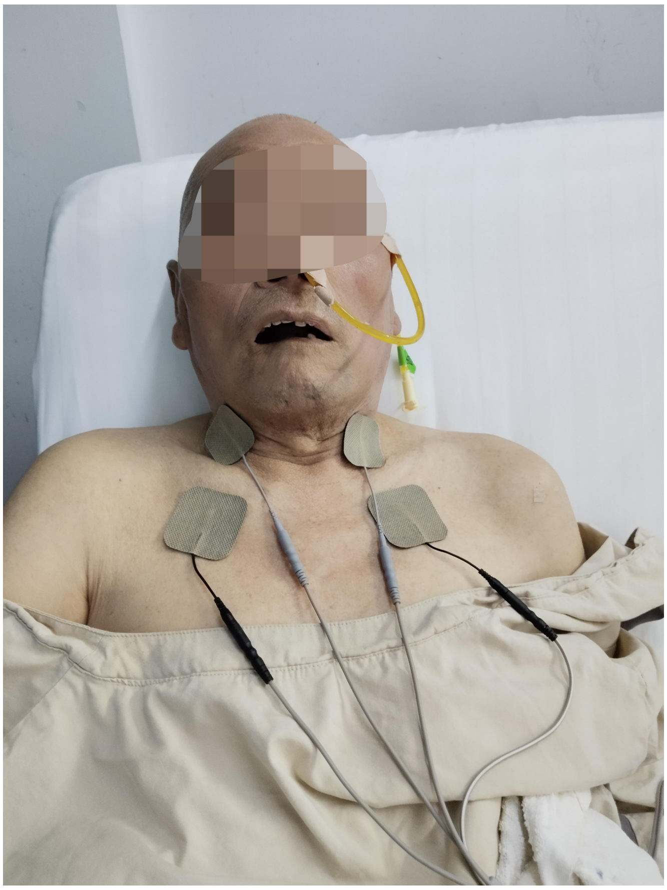

External Diaphragmatic Pacing (EDP) Training Chart for the Patient.

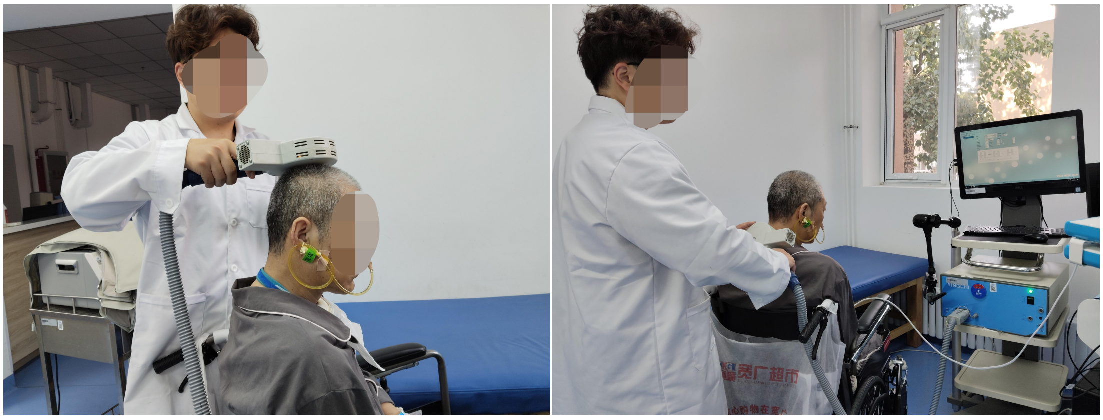

**A**: Measurement of Motor Evoked Potential in Target Muscle for Determination of Stimulation Threshold

**B**: Determination of Optimal Stimulation Site in the Neck
